# Study protocol of an economic evaluation embedded in the Midwives and Obstetricians Helping Mothers to Quit Smoking (MOHMQuit) trial

**DOI:** 10.1186/s12913-023-09898-3

**Published:** 2023-09-01

**Authors:** Alison Pearce, Joanne Scarfe, Matthew Jones, Aaron Cashmore, Andrew Milat, Larisa Barnes, Megan E. Passey

**Affiliations:** 1 The Daffodil Centre, a joint venture between Cancer Council NSW and The University of Sydney, Sydney, Australia; 2 Sydney School of Public Health, Faculty of Medicine and Health, The University of Sydney, Sydney, Australia; 3 Centre for Academic Primary Care, Unit of Lifespan and Population Health, School of Medicine, University of Nottingham, Nottingham, UK; 4 Centre for Epidemiology and Evidence, NSW Ministry of Health, Sydney, Australia; 5 University Centre for Rural Health, The University of Sydney, Sydney, Australia

**Keywords:** Cost-effective, Economic evaluation, Smoking, Pregnancy, Smoking cessation support, Tobacco, Implementation trial

## Abstract

**Background:**

Tobacco smoking during pregnancy is the most important preventable risk factor for pregnancy complications and adverse birth outcomes and can have lifelong consequences for infants. Smoking during pregnancy is associated with higher healthcare costs related to birth complications and during childhood. Psychosocial interventions to support pregnant women to quit are effective, yet provision of smoking cessation support has been inconsistent. The Midwives and Obstetricians Helping Mothers to Quit Smoking (MOHMQuit) intervention provides systems change, and leadership and clinician elements, to support clinicians to help women stop smoking in pregnancy. There have been few long-term analyses conducted of the cost-effectiveness of smoking cessation interventions for pregnant women that target healthcare providers. This protocol describes the economic evaluation of the MOHMQuit trial, a pragmatic stepped-wedge cluster-randomised controlled implementation trial in nine public maternity services in New South Wales (NSW), Australia, to ascertain whether MOHMQuit is cost-effective in supporting clinicians to help women quit smoking in pregnancy compared to usual care.

**Methods:**

Two primary analyses will be carried out comparing MOHMQuit with usual care from an Australian health care system perspective: i) a within-trial cost-effectiveness analysis with results presented as the incremental cost per additional quitter; and ii) a lifetime cost-utility analysis using a published probabilistic decision analytic Markov model with results presented as incremental cost per quality-adjusted life-year (QALY) gained for mother and child. Patient-level data on resource use and outcomes will be used in the within-trial analysis and extrapolated and supplemented with national population statistics and published data from the literature for the lifetime analysis.

**Discussion:**

There is increasing demand for information on the cost-effectiveness of implementing healthcare interventions to provide policy makers with critical information for the best value for money within finite budgets. Economic evaluation of the MOHMQuit trial will provide essential, policy-relevant information for decision makers on the value of evidence-based implementation of support for healthcare providers delivering services for pregnant women.

**Trial registrations:**

ACTRN12622000167763, registered 2 February 2022.

## Background

Tobacco smoking during pregnancy is the most important preventable risk factor for pregnancy complications and adverse birth outcomes [1]. Smoking during pregnancy increases the risk of adverse infant outcomes, including stillbirth, preterm birth, and low birth weight [1]. Maternal death is five times more likely in women who smoke during pregnancy than those who do not [1]. Smoking during pregnancy can have lifelong consequences for infants. Low birth weight is associated with childhood respiratory infections, asthma, high blood pressure, heart disease, type 2 diabetes [2–4], and being overweight or obese as a child [5–7] or adult [8, 9]. Exposure to secondhand smoke may occur during pregnancy [10, 11], or through passive exposure following birth [11–13]. Both forms of exposure can contribute to a number of health conditions in children [14].

In Australia, while the rate of smoking during pregnancy has been consistently falling in the last decade, 9.2% of women reported smoking at any time during pregnancy in 2020 [15]. Of those who smoked in the first 20 weeks of pregnancy (8.8%), the majority (78%) continued to smoke after 20 weeks [15]. The gap between Aboriginal and non-Aboriginal women who smoke during pregnancy is stark, with Aboriginal women almost six times more likely to smoke at any time during pregnancy (43.4%) than non-Indigenous women (7.5%) [15]. Higher rates of smoking during pregnancy are also seen among women aged less than 20 (34%), women aged 20–24 (21%), and those in very remote areas [15].

Globally, it is estimated that healthcare expenditure attributable to smoking-related diseases totalled US$422 billion in 2012, equivalent to 5.7% of global health expenditure [16]. Australian health care expenditure attributable to smoking was estimated to be $6.8 billion in 2015–16 [17], and tobacco use is responsible for 9.3% of the total burden of disease, greater than any other contributing risk factor [18]. Health care service usage and costs are consistently found to be higher for smokers than for non-smokers [19]. For women who smoke during pregnancy, costs associated with birth complications are 66% higher than those for non-smoking women [20], and healthcare costs during childhood have also been found to be higher for infants and children of women who smoke during pregnancy, primarily due to increased in-patient hospital care [21].

Evidence from systematic reviews indicates that psychosocial interventions to support pregnant women to quit are effective [22, 23]. Evidence-based international [24] and Australian [25, 26] guidelines recommend routine, repeated smoking cessation support (SCS) for all pregnant women using brief interventions. This can be undertaken in a few minutes during routine care [24], however provision of recommended SCS to pregnant women in Australia has remained persistently poor [27, 28]. The missing link is a failure to consistently implement effective smoking cessation care for pregnant women.

Generalisable evidence of the cost-effectiveness of interventions to improve smoking cessation is necessary to inform policy change at a system-level. A recent review of the cost-effectiveness of smoking cessation interventions for pregnant women found that interventions, such as cognitive behavioural therapy and nicotine replacement therapy, for both pregnant women and the wider population may be cost-effective from both a health system and a societal perspective [29]. However, these interventions targeted the smokers themselves and, despite evidence that system change interventions for smoking cessation can also be effective [30], few studies have investigated the cost-effectiveness of systems change approaches in the general population or in maternity services [30–32].

Here we describe in detail the protocol for an economic evaluation of the MOHMQuit intervention. MOHMQuit is a systems-change intervention being tested in a pragmatic trial undertaken in a real-world setting. The intervention includes focused training for maternity service leaders and clinicians to achieve culture change and increase prioritisation of support for smoking cessation, and provides key resources to deliver effective and appropriate SCS to pregnant women [33]. The intervention is hypothesised to increase the rates of smoking cessation in pregnancy by improving the provision of guideline-recommended SCS to pregnant women through enhancing clinicians’ knowledge, skills and confidence to provide SCS to pregnant women. Evidence of the effectiveness and cost-effectiveness of smoking cessation interventions of this kind is required to implement evidence-based, health system-wide SCS innovations at scale. The aim of this economic evaluation is to explore, from an Australian healthcare system perspective, the cost-effectiveness of MOHMQuit to increase the quit rate of pregnant women who smoke compared to usual care.

## Methods/Design

### MOHMQuit trial

**Fig.1:**
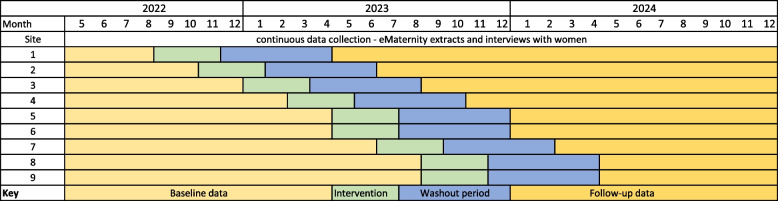
Stepped-wedge design of the MOHMQuit trial [33]

The details of the MOHMQuit intervention and the protocol for the implementation trial are described in detail elsewhere [ANZCTR #382491] [33, 34]. In summary, the MOHMQuit trial is a pragmatic stepped-wedge cluster-randomised controlled trial to be implemented in nine public hospitals providing maternity services in New South Wales (NSW). Pregnant smokers will attend a maternity service that, based on their randomised start date for the MOHMQuit intervention, has either received the MOHMQuit intervention or is still in the baseline ‘control’ period. All sites will receive the intervention, with continuous data collection occurring throughout the baseline, intervention, washout and follow-up periods for the three years of the trial [33]. The primary outcome is the 7-day point prevalence abstinence among pregnant smokers at the end of pregnancy, confirmed by salivary cotinine testing. Secondary outcomes include cost-effectiveness of the MOHMQuit intervention, rates of documentation of SCS provided, changes in clinicians’ knowledge, confidence, and attitudes to providing SCS, women’s views on SCS received, and intervention fidelity. The MOHMQuit trial is due to be completed in December 2024. See Fig. 1 for a diagram of the stepped-wedge design and timeframe.

### Economic evaluation overview

The economic evaluation will be conducted from an Australian healthcare system perspective and include costs in Australian dollars (AUD) for the year 2022. The economic evaluation will be undertaken by adapting the Economics of Smoking in Pregnancy (ESIP) Model [35, 36] to use NSW or Australian data where possible. Two primary analyses will be carried out: a) a within-trial cost-effectiveness analysis with an 8-month time horizon, with results presented as the incremental cost per additional quitter; and b) a lifetime cost-utility analysis, with results presented as incremental cost per quality-adjusted life-year (QALY) gained for mother and child, presented both separately and as a combined ‘per pregnancy’ measure of cost-effectiveness. The results of the cost-utility analysis will be assessed against a commonly used benchmark of $50,000 AUD per QALY gained [37–39]. A secondary analysis of cost–benefit over the lifetime will also be conducted.

The robustness of the cost-effectiveness and cost-utility analyses will be explored using probabilistic sensitivity analysis and expressed in a cost-effectiveness acceptability curve. Return on investment estimates will also be produced for maternal and infant health care (separately and combined) for the lifetime time horizon. Cost–benefit ratios (defined as incremental health care savings divided by incremental intervention cost) will be produced. The economic evaluation will adhere to current best practice guidelines [40, 41] and analysis will be undertaken using Microsoft Excel 2010 [42].

### Within-trial analysis

Patient-level data on resource use and outcomes will be routinely collected during the trial and used in the economic evaluation. The time horizon for the within-trial analysis is 8 months, representing the maximum time each woman is in contact with the health service, from early pregnancy to immediately after birth. No discounting of costs and outcomes is required as this time horizon is less than one year. The analysis will be undertaken based on an intention-to-treat approach, and participants lost to follow-up will be assumed to be continuing smokers.

#### Measurement and valuation of resource use (within-trial analysis)

The three resource use groups of relevance to the within-trial analysis are: delivery of the intervention; clinician and leader time to engage with the intervention; and neonatal care. A summary of the sources of measurement and valuation for intervention costs are shown in Table 1. Intervention implementation will be monitored using project management logs, training logs, eMaternity data, and participant (clinician, manager, and patient) self-report. Clinician and clinical leader time to engage with MOHMQuit activities will be collected through self-report. The child’s birth weight, date of delivery, gestational age (allowing calculation of preterm status), and whether the baby is admitted to the special care nursery or neonatal intensive care unit will be collected postpartum via the trial data management system.

**Table 1:** Measurement and valuation of intervention costs

**Cost**	**Specification**	**Source of measurement**	**Source of value (unit costs)**
**Intervention**			
Training for maternity service leaders	Time for facilitator to prepare for, travel to and from, and run the workshop Number of attendees at workshop: Most sites: 1 × 3-h workshop Larger sites: 1 × 4-h workshop Number of staff completing each training module (two modules, 30 min total) Workshop-related printing costs	Documented facilitator time Workshop attendance records Clinician and midwife training questionnaires MOHMQuit admin records	Average salary of facilitator and attendees sourced from NSW Award wages Actual costs incurred
Key resources for managers and clinical leaders	Manager/clinical leader time to review eMaternity reports monthly, develop and maintain champions, complete the audit and action planning tool annually, develop local care pathways and other relevant actions (as required)	3-month clinician questionnaire to capture which components have been completed 6-month qualitative interviews to capture time involved in MOHMQuit program overall, and opportunity cost	Average salary of attendees sourced from NSW Award wages
Training for clinicians	Time for facilitator to prepare for, travel to and from, and run the workshop Number of attendees at workshop: • Midwives and Aboriginal Health Workers: Full day (7.5- h) training • Obstetricians and Obstetric trainees: 2- h training Number of staff completing each training module (two modules, 30 min total) Workshop-related printing costs	Facilitator documentation Workshop attendance records Clinician and midwife training questionnaires MOHMQuit admin records	Average salary of attendees sourced from NSW Award wages Actual costs incurred
Community of practice meetings	1-h online meeting one month following implementation at each site	Meeting attendance records	Average salary of attendees sourced from NSW Award wages

Unit costs will be obtained from routine sources, including NSW Award Wage scales (to value time of MOHMQuit implementation team and clinician/leader engagement with the intervention), trial data management system (for consumables), and IHACPA (Independent Health and Aged Care Pricing Authority) National Hospital Cost Data Collection (for neonatal costs) [43]. Both mean and median costs will be presented [44]. Where appropriate, mean cost estimates will be used with confidence intervals generated through bootstrapping. All costs will be presented in AUD and updated to a standard reference year (2022) for analysis. Unit cost information will be combined with the resource use data to estimate the total cost per pregnant smoker who received maternity care either with or without the MOHMQuit intervention. These per person total costs will be aggregated to estimate the overall total cost of MOHMQuit and standard care and subsequently the average cost per pregnant smoker for each. Costs associated with providing the intervention will be reported separately. The proportion of leaders, clinicians and patients who participate in the MOHMQuit intervention, and the proportion of women who report receiving SCS, will be presented. Clinician time to implement SCS strategies is incorporated into standard care and will not have a cost associated with it.

#### Measurement and valuation of benefit (within-trial analysis)

The primary benefit of the intervention will be measured by comparing the 7-day point prevalence abstinence at the end of pregnancy, confirmed by salivary cotinine testing, among women who report current smoking or quitting since becoming pregnant at antenatal booking in the intervention period to the control (baseline) period. This will inform the incremental cost per additional quitter analysis. Seven-day point prevalence abstinence with biochemical verification is recommended as an outcome measure [45, 46], and is commonly used in pregnancy smoking cessation trials, as longer timeframes are not relevant to benefits to the foetus [47, 48]. It is recognised that this outcome has limitations, in that it is an intermediate outcome and not a measure of health. However, collecting broader outcomes is not practicable in the context of this trial as data will only be collected directly from women in postpartum interviews. Additionally, the myriad physical and social changes that occur with birth and in the early postnatal period [49, 50] make it difficult to assess whether changes in quality of life outcomes would be due to smoking cessation, the postpartum stage, or other factors.

#### Analysis and sensitivity analysis (within-trial analysis)

The cost-effectiveness analysis will use the estimates of cost and effect as described above to estimate an incremental cost-effectiveness ratio (ICER) of cost per quitter. Where significant levels of missing data occur (5% or greater of the observations), approaches to account for missingness will be undertaken, including multiple imputation to account for data missing at random or missing completely at random [51] and other relevant best practice approaches [52].

Sensitivity analyses will examine uncertainty around the primary endpoint, costs, the fidelity of implementation, and the impact of economies of scale if the intervention was rolled out at the population level. Sensitivity analysis will be undertaken using non-parametric bootstrapping to provide the confidence ellipse, which reflects the uncertainty in the estimate of the ICER. The ellipse provides a region on the cost-effectiveness plane that should contain 95% of the uncertainty [53]. Uncertainty regarding the cost-effectiveness of the intervention will be summarised using a cost-effectiveness acceptability curve (CEAC) and will represent the likelihood of the intervention being cost-effective at a range of ceiling willingness to pay thresholds for an additional woman quitting.

#### Lifetime model

The time horizon for the lifetime economic model is lifetime of both the mother and the child. Costs and effects will be discounted at 5% annually in line with recommendations from NSW Treasury [54]. While the within-trial analysis considers the costs and effects of the pregnant women and neonatal costs only across the eight months follow-up, the lifetime model expands the evaluation to include the healthcare costs and health outcomes of smoking behaviour and any changes across the mother and child’s lifetime. This includes the impacts of the child’s exposure to secondhand smoke, and that they are more likely to smoke themselves, increasing the likelihood of future smoking-related diseases [55].

##### Model structure (lifetime model)

To conduct the lifetime analysis we will use an adapted version of a published decision analytic model – the ESIP model, modified for Australian populations [35, 36]. ESIP predicts the impact that smoking both in pregnancy and after can have on the lifetime healthcare costs and health outcomes for the mother and her offspring. Using ESIP enables us to expand the evaluation to include the benefits of stopping smoking which occur beyond the short time horizon of the within-trial cost-effectiveness analysis.

**Fig. 2:**
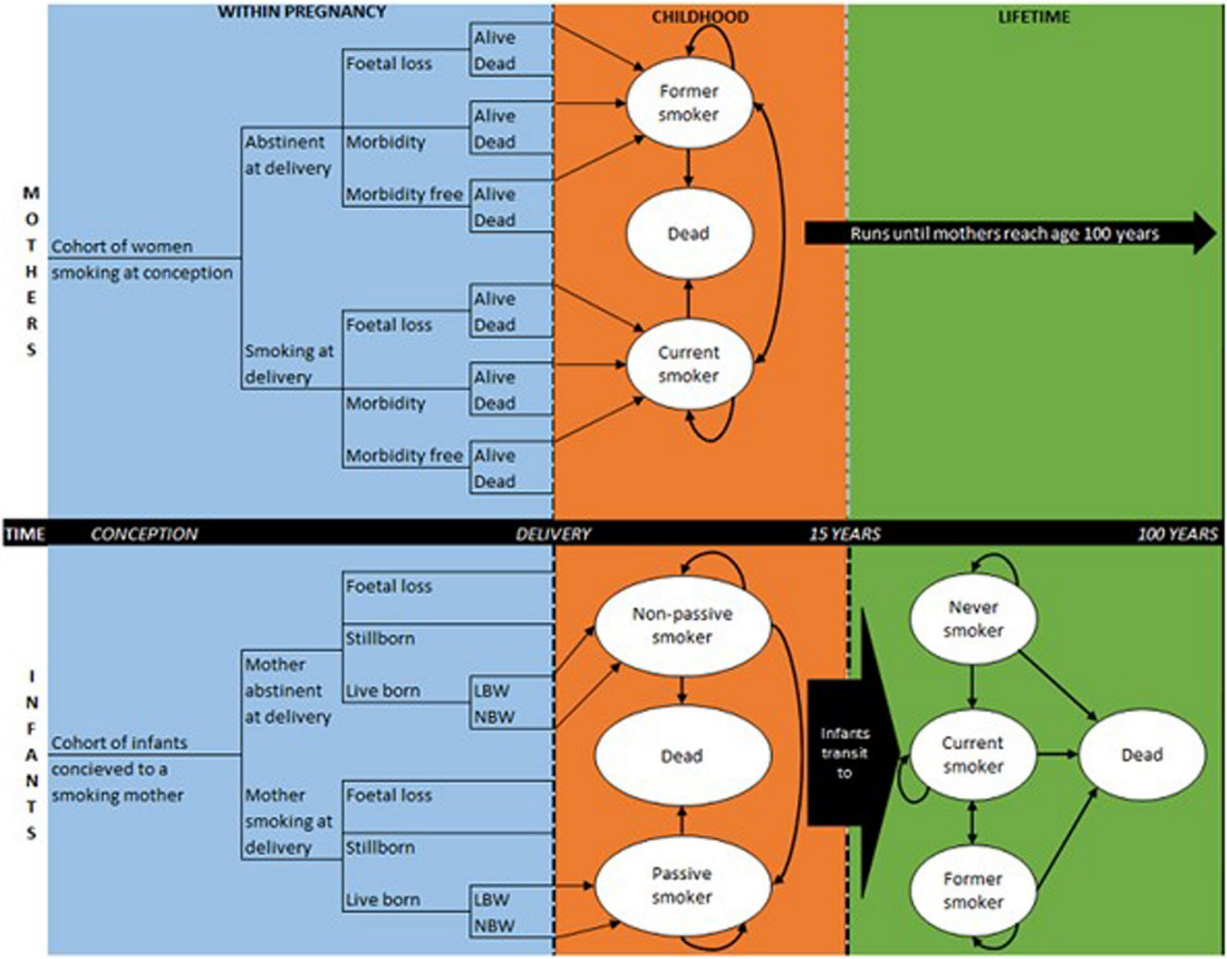
Original ESIP model structure [36] LBW: low birth weight; NBW: normal birth weight

The structure of the original ESIP model is shown in Fig. 2. The model is divided into mother and infant, and within-pregnancy (outcomes and costs during pregnancy), childhood (outcomes and costs for the infant from birth to 15 years) and lifetime (lifetime outcomes and costs for both mother and infant) time periods. The mother’s component of the model will include the costs of SCS, smoking-related diseases (coronary heart disease [CHD], chronic obstructive pulmonary disease [COPD], lung cancer and stroke), data on quality of life, relapse rates and transition to smoking-related diseases if the mother relapses. The childhood component of the model will include costs associated at birth for children with smoking mothers if relevant (for premature birth and low birth weight newborns), outcomes related to secondhand smoke exposure in the home, quality of life, smoking uptake rates for children of smokers and associated smoking-related morbidities (asthma, CHD, COPD, lung cancer and stroke). Resource use and outcomes collected during the trial will be extrapolated and supplemented with published data from the literature.

ESIP will be modified for the Australian population by using Australian and NSW data sources where possible. For example, the Cancer Institute NSW Smoking and Health and Tobacco Tracking Surveys (maternal post-partum smoking behaviour and maternal and partner lifetime smoking behaviour), NSW Perinatal Data Collection (maternal morbidities and outcomes, and infant birth outcomes), Australian Bureau of Statistics National Health Survey (prevalence of morbidities among general population [CHD, stroke, COPD, lung cancer, childhood asthma]), Australian Institute of Health and Welfare National Drug Strategy Household Survey (infant exposure to secondhand smoke in the home).

#### Analysis and sensitivity analysis (lifetime model)

The lifetime analysis results will be presented as incremental cost per QALY gained for both the mother and child, separately and combined. Cost–benefit analysis ratios (defined as incremental health-care savings divided by incremental intervention cost) will be produced for the lifetime time horizon. Deterministic and probabilistic sensitivity analyses will be undertaken for the range of areas of uncertainty (informed by the 95% confidence interval or standard error of the mean input value where available) to explore underlying model assumptions. One-way sensitivity analysis will be undertaken to demonstrate the impact of varying specific inputs for: self-reported outcomes; alternative discount rates (3% and 7%); varying relapse rates and changing adherence rates. Two-way sensitivity analysis will be carried out on variables found to substantially increase the ICER in one-way sensitivity analysis, and those which are correlated. Probabilistic sensitivity analysis will be undertaken using model parameter distributions for ESIP’s 390 input variables. Methods used for fitting distributions have been described elsewhere [35]. Ten thousand Monte Carlo simulations will be performed, and cost-effectiveness acceptability curves produced.

## Discussion

While many pregnant women are highly motivated to quit smoking [56], they face significant challenges including a lack of effective support from clinicians [57]. This study represents one of the few analyses conducted of the cost-effectiveness of smoking cessation interventions that target antenatal healthcare providers for pregnant women and conducted under ‘real world’ conditions. There is increasing demand for evidence of cost-effectiveness of implementation of healthcare interventions to provide policy makers with critical information for the best value-for-money spend on finite budgets. Randomised controlled trials of the clinical effectiveness of interventions and their implementation can provide good opportunities to conduct an economic evaluation alongside the trial, provided the appropriate steps are taken from the outset to ensure that the design of the trial is fit-for-purpose [51]. Decreasing antenatal smoking to reduce the effects on pregnancy and newborn outcomes is a government priority [58, 59]. Evidence of the effectiveness and cost-effectiveness of smoking cessation interventions is required to implement evidence-based, health system-wide SCS innovations. This study will provide evidence of MOHMQuit’s costs and benefits to inform decisions on scalability.

The MOHMQuit trial economic evaluation has been planned using available national and international guidelines for conducting economic evaluations [51, 60], promoting greater transparency in the methods undertaken and increasing the rigor and validity of the findings. While it will draw on the MOHMQuit trial results for model inputs, the economic evaluation will adopt its own methods and analysis and results will be reported separately from the main trial without duplicating information. The ESIP model provides a comprehensive approach to estimating costs, outcomes, and long-term cost-effectiveness and cost-utility, of smoking cessation interventions in pregnancy [35]. Its ability to provide common outcome measures (for example, incremental cost per QALY) allow comparisons between smoking cessation and other healthcare interventions. Where possible, ESIP will be populated with NSW and Australian data, however initial investigations have found that local level data may not be available for all model parameters. The analysis might be strengthened through adaptation of the ESIP model to allow for comparison between population sub-groups with higher rates of smoking in pregnancy. The study will provide essential policy-relevant information for decision makers on the value of evidence-based implementation of support for antenatal healthcare providers delivering SCS for pregnant women.

## Data Availability

Not applicable.

## Abbreviations

AUD: Australian dollars
CEAC: Cost-effectiveness acceptability curve
CHD: Coronary heart disease
COPD: Chronic obstructive pulmonary disease
ESIP: Economics of Smoking in Pregnancy
ICER: Incremental cost-effectiveness ratio
MOHMQuit: Midwives and Obstetricians Helping Mothers to Quit Smoking
NSW: New South Wales
QALY: Quality-adjusted life-year
SCS: Smoking cessation support

## References

[1] Australian Institute of Health and Welfare. Australia’s mothers and babies: Maternal deaths - web article. 2021. Cited 13 Jul 2022. Available from: https://www.aihw.gov.au/reports/mothers-babies/maternal-deaths-australia.

[2] Lumley J, Chamberlain C, Dowswell T, Oliver S, Oakley L, et al. Interventions for promoting smoking cessation during pregnancy. Cochrane Database Syst Revs. 2009;3(CD001055). Available from: https://www.ncbi.nlm.nih.gov/pubmed/19588322. 10.1002/14651858.CD001055.pub3 PMC4090746 19588322

[3] Barker D, Eriksson J, Forsen T, Osmond C (2002). Fetal origins of adult disease: Strength of effects and biological basis. Int J Epidemiol.

[4] Barker D (1998). In utero programming of chronic disease. Clin Sci.

[5] Oken E, Levitan E, Gilman M (2008). Maternal smoking during pregnancy and child overweight: systematic review and meta-analysis. Int J Obes.

[6] Ino T (2010). Maternal smoking during pregnancy and offspring obesity: Meta-analysis. Pediatr Int.

[7] Riedel C, Schonberger K, Yang S, Koshy G, Chen Y (2014). Parental smoking and childhood obesity: higher effect estimates for maternal smoking in pregnancy compared with paternal smoking - a meta-analysis. Int J Epidemiol.

[8] Kataria Y, Gaewsky L, Ellervik C (2019). Prenatal smoking exposure and cardio-metabolic risk factors in adulthood: a general population study and a meta-analysis. Int J Obes.

[9] Magalhaes E, Sousa B, Lima N, Horta B (2019). Maternal smoking during pregnancy and offspring body mass index and overweight: a systematic review and meta-analysis. Cad Saude Publica.

[10] US Department of Health and Human Services. How tobacco smoke causes disease: the biology and behavioral basis for smoking-attributable disease. A report of the US Surgeon General. Atlanta, Georgia: US Department of Health and Human Services, Centers for Disease Control and Prevention, National Center for Chronic Disease Prevention and Health Promotion, Office on Smoking and Health. 2010. Available from: https://www.ncbi.nlm.nih.gov/books/NBK53017/.

[11] US Department of Health and Human Services. The health consequences of involuntary exposure to tobacco smoke: a report of the Surgeon General. Atlanta, Georgia: US Department of Health and Human Services, Centers for Disease Control and Prevention, Coordinating Center forHealth Promotion, National Center for Chronic Disease Prevention and Health Promotion, Office on Smoking and Health. 2006. Available from: http://www.cdc.gov/tobacco/data_statistics/sgr/sgr_2006/index.htm.

[12] US Department of Health and Human Services. Women and smoking. A report of the US Surgeon General. Atlanta, Georgia: US Department of Health and Human Services, Centers for Disease Control and Prevention, National Center for Chronic Disease Prevention and Health Promotion, Office on Smoking and Health. 2001. Available from: https://www.cdc.gov/tobacco/data_statistics/sgr/2001/index.htm.

[13] British Medical Association Board of Science and Education and Tobacco Control Resource Centre, Smoking and Reproductive Life. The impact of smoking on sexual, reproductive and child health. British Medical Association. 2004. Available from: https://www.rauchfrei-info.de/fileadmin/main/data/Dokumente/Smoking_ReproductiveLife.pdf.

[14] Campbell M, Greenhalgh E, Ford C, Winstanley M. Chapter 4.7: Estimates of morbidity and mortality attributable to secondhand smoke. In: Tobacco in Australia: Facts and issues. Melbourne: Cancer Council Victoria. 2019. Available from: http://www.tobaccoinaustralia.org.au/chapter-4-secondhand/4-7-estimates-of-morbidity-and-mortality.

[15] Australian Institute of Health and Welfare. Australia’s Mothers and Babies. Canberra: Australian Institute of Health and Welfare. Report No.: Cat. no. PER 101. 2022. Cited 10 Nov 2022. Available from: https://www.aihw.gov.au/reports/mothers-babies/australias-mothers-babies/contents/antenatal-period/smoking-during-pregnancy.

[16] Goodchild M, Nargis N, Tursan DE (2018). Global economic cost of smoking-attributable diseases. Tob Control.

[17] Whetton S, Tait R, Scollo M, Banks E, Chapman J. Identifying the social costs of tobacco use to Australia in 2015/16. Perth, Western Australia: The National Drug Research Institute at Curtin University. 2019. Available from: http://ndri.curtin.edu.au/NDRI/media/documents/publications/T273.pdf.

[18] Australian Institute of Health and Welfare 2019. Burden of Tobacco Use in Australia: Australian Burden of Disease Study 2015. Australian Burden of Disease series no. 21. Cat. no. BOD 20. Canberra: AIHW

[19] Scollo M, Greenhalgh E. The costs and benefits of smoking to the Australian economy. In: Tobacco in Australia: Facts and Issues. Melbourne: Cancer Council Victoria. 2021. Available from: https://www.tobaccoinaustralia.org.au/chapter-17-economics/17-2-the-costs-of-smoking.

[20] Centers for Disease Control and Prevention. Medical care expenditures attributable to cigarette smoking during pregnancy. United States: Centers for Disease Control and Prevention. Morb Mortal Wkly Rep. 1997;46(44). cited 10 Nov 2022. Available from: https://www.cdc.gov/mmwr/PDF/wk/mm4644.pdf. 9370226

[21] Vaz L, Jones M, Szatkowski L, Tata L, Petrou S (2018). Estimating the healthcare costs of children born to pregnant smokers in England: Cohort study using primary and secondary healthcare data. Addiction.

[22] Chamberlain C, O’Mara-Eves A, Porter J, Coleman T, Perlen S, Thomas J, et al. Psychosocial interventions for supporting women to stop smoking in pregnancy. John Wiley & Sons, Ltd.; 2017. Cochrane Database Syst Rev. Available from: https://www.cochranelibrary.com/cdsr/doi/10.1002/14651858.CD001055.pub5/full. 10.1002/14651858.CD001055.pub5 PMC6472671 28196405

[23] Bar-Zeev Y, Bonevski B, Lim L, Twyman L, Skelton E, Gruppetta M (2019). Improving health providers smoking cessation care in pregnancy: A systematic review and meta-analysis. Addict Behav.

[24] U.S. Preventive Services Task Force. Counseling and interventions to prevent tobacco use and tobacco-caused disease in adults and pregnant women: U.S. Preventive Services Task Force reaffirmation recommendation statement. Ann Intern Med. 2009;150(8):551–5. 10.7326/0003-4819-150-8-200904210-00009. 10.7326/0003-4819-150-8-200904210-00009 19380855

[25] NSW Ministry of Health. Clinical Guidelines for the Management of Substance Use During Pregnancy, Birth and the Postnatal Period - Professionals. Sydney: NSW Ministry of Health. 2014. Available from: https://www.health.nsw.gov.au/aod/professionals/Pages/substance-use-during-pregnancy-guidelines.aspx.

[26] The Royal Australian College of General Practitioners (RACGP). Supporting smoking cessation: a guide for health professionals. East Melbourne, Victoria: RACGP; 2019.

[27] Perlen S, Brown S, Yelland J (2013). Have Guidelines About Smoking Cessation Support in Pregnancy Changed Practice in Victoria, Australia?. Birth.

[28] Passey M, Sanson-Fisher R (2015). Provision of Antenatal Smoking Cessation Support: A Survey With Pregnant Aboriginal and Torres Strait Islander Women. Nicotine Tob Res.

[29] Department of Health. 2018. Clinical Practice Guidelines: Pregnancy Care. Economic Analyses. Canberra: Australian Government Department of Health.

[30] Thomas D, Abramson M, Bonevski B, George J. System change interventions for smoking cessation. Cochrane Databaase of Systematic Reviews . 2(Art. No.: CD010742). Cited 22 Mar 2023. Available from: https://www.cochranelibrary.com/cdsr/doi/10.1002/14651858.CD010742.pub2/full. 10.1002/14651858.CD010742.pub2 PMC6464284 28185257

[31] Ontario Agency for Health Protection and Promotion (Public Health Ontario), Berenbaum E, Keller-Olaman S, Watson K, Longo C. Economic Benefits of Smoking Cessation Intervention: Rapid Review [Internet]. Queen’s Printer for Ontario. 2019. Cited 22 Mar 2023. Available from: https://www.publichealthontario.ca/-/media/documents/r/2020/rapid-review-economic-benefits-smoking-cessation.pdf?la=en.

[32] Greenhalgh E, Hurley S, Lal A. 17.4 Economic evaluations of tobacco control interventions. In Greenhalgh EM, Scollo MM and Winstanley MH (editors). Tobacco in Australia: Facts and Issues. (Melbourne: Cancer Council Victoria). 2020. Cited 22 Mar 2023. Available from: https://www.tobaccoinaustralia.org.au/chapter-17-economics/17-4-economic-evaluations-of-tobacco-control-interventions.

[33] Barnes L, Longman J, Adams C, Paul C, Atkins L, Bonevski B (2022). The MOHMQuit (Midwives and Obstetricians Helping Mothers to Quit Smoking) Trial: protocol for a stepped-wedge implementation trial to improve best practice smoking cessation support in public antenatal care services. Implement Sci.

[34] Passey M, Adams C, Paul C, Atkins L, Longman J (2021). Improving implementation of smoking cessation guidelines in pregnancy care: development of an intervention to address system, maternity service leader and clinician factors. Implement Sci Commun.

[35] Jones M, Smith M, Lewis S, Parrott S, Coleman T (2019). A dynamic, modifiable model for estimating cost-effectiveness of smoking cessation interventions in pregnancy: application to an RCT of self-help delivered by text message. Addiction.

[36] Division of Primary Care, University of Nottingham. Tobacco and Alcohol Research: The Economics of Smoking in Pregnancy (ESIP) Model. Cited 8 Dec 2022. Available from: https://www.nottingham.ac.uk/research/groups/tobaccoandalcohol/smoking-in-pregnancy/esip/index.aspx.

[37] Wang S, Gum D, Merlin T (2018). Comparing the ICERs in Medicine Reimbursement Submissions to NICE and PBAC-Does the Presence of an Explicit Threshold Affect the ICER Proposed?. Value Health J Int Soc Pharmacoeconomics Outcomes Res.

[38] Zhang K, Garau M. International Cost-Effectiveness Thresholds and Modifiers for HTA Decision Making. London: Office of Health Economics. 2020. Available from: https://www.ohe.org/publications/international-cost-effectiveness-thresholds-and-modifiers-hta-decision-making.

[39] McDougall J, Furnback W, Wang B, Mahlich J (2020). Understanding the global measurement of willingness to pay in health. J Mark Access Health Policy.

[40] Husereau D, Drummond M, Petrou S, Carswell C, Moher D, Greenberg D (2013). Consolidated Health Economic Evaluation Reporting Standards (CHEERS) statement. BMJ.

[41] Australian Government Department of Health. PBAC Guidelines. Cited 9 Dec 2021. Available from: https://pbac.pbs.gov.au/.

[42] Plus MOP (2010). Micrsoft Office Excel 2010.

[43] IHPA. National Hospital Cost Data Collection: Public Hospitals Cost Report, Round 18 (Financial year 2013–14). 2016. Cited 28 Feb 2022. Available from: https://www.ihpa.gov.au/publications/australian-public-hospitals-cost-report-2013-2014-round-18.

[44] Thompson S, Barber J (2000). How should cost data in pragmatic randomised trials be analysed?. BMJ.

[45] Piper M, Bullen C, Krishnan-Sarin S, Rigotti N (2020). Defining and measuring abstinence in clinical trials of smoking cessation interventions: An updated review. Nicotine Tob Res.

[46] Tobacco Use and Dependence Guideline Panel. Treating Tobacco Use and Dependence: 2008 Update. Rockville (MD): US Department of Health and Human Services; 2008. Available from: https://www.ncbi.nlm.nih.gov/books/NBK63952/.

[47] Heil S, Higgins S, Bernstein I, Solomon L, Rogers R, Thomas C (2008). Effects of voucher-based incentives on abstinence from cigarette smoking and fetal growth among pregnant women. Addiction Abingdon Engl.

[48] Pollak K, Lyna P, Gao X, Noonan D, Bejarano Hernandez S, Subudhi S (2020). Efficacy of a texting program to promote cessation among pregnant smokers: A randomized control trial. J Soc Res Nicotine Tob.

[49] Jeong YJ, Kim H, Kim J (2021). Factors influencing quality of life in early postpartum women. Int J Environ Res Public Health.

[50] Verbiest S, Tully K, Simpson M (2018). Elevating mothers’ voices: recommendations for improved patient-centered postpartum. J Behav Med.

[51] Ramsey S, Willke R, Glick H, Reed S (2015). Cost-effectiveness analysis alongside clinical trials II: An ISPOR Good Research Practices Taskforce Report. Value Health J Int Soc Pharmacoeconomics Outcomes Res.

[52] Faria R, Gomes M, Epstein D, White I (2014). A guide to handling missing data in cost-effectiveness analysis conducted within randomised controlled trials. Pharmacoeconomics.

[53] Black W (1990). The CE plane: A graphic representation of cost-effectiveness. Int J Soc Med Decis Mak.

[54] NSW Treasury. NSW Government Guide to Cost Benefit Analysis. NSW Treasury. 2023. Report No.: TPG23–08. Cited 24 May 2023. Available from: https://www.treasury.nsw.gov.au/sites/default/files/2023-04/tpg23-08_nsw-government-guide-to-cost-benefit-analysis_202304.pdf.

[55] Leonardi-Bee J, Jere M, Britton J (2011). Exposure to parental and sibling smoking and the risk of smoking uptake in childhood and adolescence: A systematic review and meta-analysis. Thorax.

[56] McBride C, Emmons K, Lipkus I (2003). Understanding the potential of teachable moments: the case of smoking cessation. Health Educ Res.

[57] Flemming K, Graham H, McCaughan D, Angus K, Bauld L (2015). The barriers and facilitators to smoking cessation experienced by women’s partners during pregnancy and the post-partum period: A systematic review of qualitative research. BMC Public Health.

[58] Commonwealth of Australia. National Tobacco Strategy 2023–2030. Department of Health and Aged Care. Report No.: Publications number 12710. Cited 10 May 2023. Available from: https://www.health.gov.au/sites/default/files/2023-05/national-tobacco-strategy-2023-2030.pdf.

[59] NSW Health. Reducing the effects of smoking and vaping on pregnancy and newborn outcomes. NSW Government. 2022. Report No.: PD2022_050. Cited 10 May 2023. Available from: https://www1.health.nsw.gov.au/pds/ActivePDSDocuments/PD2022_050.pdf.

[60] Australian Government Department of Health. Pharmaceutical Benefits Scheme: Manual of resource items and their associated costs. Australian Government Department of Health; 2016. Cited 26 Apr 2021. Available from: https://www.pbs.gov.au/pbs/industry/useful-resources/manual.

